# Growth hormone–deficient Ames dwarf mice resist sarcopenia and exhibit enhanced endurance running performance at 24 months

**DOI:** 10.1007/s11357-025-01630-9

**Published:** 2025-03-26

**Authors:** Matthew J. Johnston, Sharlene G. Rakoczy, LaDora V. Thompson, Holly M. Brown-Borg

**Affiliations:** 1 Biomedical Sciences Department, University of North Dakota, 504 Hamline St., Grand Forks, ND 58203 USA; 2 Department of Physical Therapy, Boston University, Boston, MA USA

**Keywords:** Growth hormone, Sarcopenia, Muscle, Aging, Exercise

## Abstract

Ames dwarf mice (*df/df*) live 50% longer than normal littermates due to a genetic defect in growth hormone (GH) signaling. The enhanced longevity of Ames dwarfs has been studied extensively in an endocrinological context of cellular metabolism and increased resistance to oxidative stress (Bartke. World J Mens Health 37(1):19, 8; Bartke 2; BartkeJ Am Aging Assoc 23(4):219, 10; Bartke. World J Mens Health 39(3):454-465, 11; Brown-Borg et al. Nature 384(6604):33-33, 1; Masternak et al. 2018). However, the skeletal muscle system is relatively unexplored, the quality of which dictates metabolic homeostasis, permits movement and exercise, and exerts paracrine effects on other organs (Delmonico and Beck Am J Lifestyle Med 11(2):167-181, 25; Evans et al. GeroScience 46(1):183, 26; Kim and Kim. Endocrinol Metab (Seoul) 35(1):1-6, 15; Masternak et al. 2018). Here, we characterize the fitness capacity and skeletal muscle morphology of Ames mice to determine if previously established longevous effects of GH deficiency extend to skeletal muscle tissue. Mutually exclusive, age-matched cohorts of male Ames mice and wildtype controls performed grip strength, rotarod, and endurance running experiments over 6 months. The largest difference in physical performance was observed in endurance running capacity, where dwarf mice outperformed wildtype controls increasingly with age. Tibialis anterior (TA) muscles were evaluated for myofiber size, quality, and environment. Ames mice show reduced myofiber cross-sectional area (CSA) paired with increased myofibers per muscle. Dwarf myofiber populations are less heterogenous in size and seemingly resist sarcopenia, as skeletal muscle from aged individuals shows youthful morphological resemblance in mean myofiber CSA, size frequency distribution, and presence of fibrotic tissue. Declines in fitness performance and myofiber integrity were observable in age-matched wildtype controls. Utilizing an established longevity model to investigate skeletal muscle function and morphology is a novel approach to gaining insight into the seemingly inverse relationship between GH signaling and mammalian longevity.

**Supplementary Information:**

The online version contains supplementary material available at 10.1007/s11357-025-01630-9.

## Introduction

Ames dwarf mice (*Prop1*^*df*^*/Prop1*^*df*^) have been studied in the context of aging since 1996, when it was reported that they outlived their normal-sized siblings by a full year, or 50% on average [1]. The Ames dwarf phenotype presents mice that are half-sized and blunt-nosed yet share an otherwise identical genetic background with their normal siblings, as well as matching maternal and living conditions [1–6]. Ames dwarfs are characterized by defect synthesis of growth hormone (GH), prolactin, and thyrotropin due to a homozygous recessive mutation at the Prophet of Pit-1 *(Prop-1)* locus [1, 2, 7]. Prop-1 is a transcription factor responsible for the differentiation of somatotrophic, lactotrophic, and thyrotrophic cell types within the anterior pituitary gland. Mutant Prop-1 results in pituitary hormone deficiency that induces a secondary deficiency in insulin-like growth factor 1 (IGF-1), as hepatic IGF-1 synthesis is almost exclusively triggered by GH action [8]. Circulating IGF-1 mediates the growth-promoting action of GH on bone and muscle tissue, with GH alone exerting more metabolic influence. The combined inactivity of GH/IGF-1 signaling in Ames dwarfs induces evolutionary trade-offs of growth retardation and limited fecundity in exchange for enhanced health span and longevity. Attenuation of GH signaling results in several metabolic alterations, as Ames dwarfs exhibit reductions in insulin secretion, plasma glucose levels, and protein production, paired with improved insulin sensitivity, antioxidant defenses, and stress resistance; all proposed to constitute their longevity [1–5, 7–11]. Ames mice exhibit reduced protein synthesis and cellular proliferation via reduced PI3-Kinase/Akt signaling and therefore decreased activation of its downstream target, mTORC1 [8, 11]. This results in a cellular population that at matched chronological age has undergone less replication and protein proliferation, thereby maintaining more “youthful” replicatory, apoptotic, and stress-resistant cellular capabilities. This idea is supported by previous work done in hepatic cells of Ames mice that showed an increase in pro-apoptotic proteins [12], constituting improved cellular quality control that rids dysfunctional cells via apoptosis at high efficacy due to absence of growth signals that may otherwise influence damaged cells to continue to proliferate. Furthermore, improved clearance of reactive oxygen species (ROS) through altered antioxidant enzyme levels has been confirmed across various cell types of Ames dwarfs [4, 6, 13, 14]. Whether the mechanisms listed above are synonymous with other GH-deficient species is a topic of current research but outside the scope of this study.

Although reports exist on the various endocrinological aspects of this mutation, the consequence of the lack of these hormones on skeletal muscle morphology and function is rather limited. Skeletal muscle is a major metabolic organ due to its large mass and its significant role in regulating blood glucose levels through insulin-mediated glucose uptake, making it a key player in overall energy homeostasis and amino acid storage [15, 16]. Previous skeletal muscle work in Ames dwarfs involved assessing lean body mass and having mice perform swimming tests for the subsequent observation of ROS production and clearance, pointing to improved antioxidant defense compared to wildtype controls [14]. Furthermore, the physical performance of Ames dwarf mice and GH receptor KO (GHR-KO) mice has been previously reported as greater than that of hormonally normal littermates across wire hang (strength), inverted screen (motor coordination), and inclining rod (balance) tests [17]. This study employed a scoring system based on deviations from a perfect score of 1—that being the score for a healthy, young mouse [17]. This marked the first paradoxical description of somatotropic signaling-deficient mice exhibiting superior performance in physical tests that would seemingly favor increased muscle mass and bone density. However, the definition of fitness might have been compromised by the “ceiling effect” of perfect performance (i.e., a score of 1), leaving true quantitative measures of physical capacity absent. Furthermore, comprehensive physical assessment should include multiple rounds of testing across a robust period of time to better establish trends dependent and exclusive of age groups—which remains undocumented to this point. Despite this, prior inkling that growth-hormone deficient dwarf mice exhibit improved physical performance provided an exciting analog for our base of work. Administering physical tests analogous to human clinical frailty assessment to large, mutually exclusive age groups of Ames dwarf mice and wildtype controls offers a novel approach. We hypothesize that long-lived Ames dwarf mice will exhibit altered skeletal muscle morphology and attenuation of age-related declination in both physical fitness capacity and myofiber integrity. In our study, we aim to further clarify the paradoxical relationship between anabolic-deficient mice and their physical fitness capacity, addressing claims about both enhancements and impairments in both relative and absolute physical performance.

## Methods

### Animals

Ames dwarf mice were derived from a closed colony with a heterogeneous background. Homozygous (*df/df*) or heterozygous (*df/+*) dwarf males were mated with carrier females (*df/+)* to generate dwarf and wildtype mice at the University of North Dakota. All mice used in the study were males due to availability, colony status, and constraints of generating dwarf mice of diverse age groups. Ames dwarf and wildtype control mice were chronologically age-matched across three groups. Groups were divided into young (3–7 months old), middle-aged (8–12 months old), and aged (18+ months old) mice at the beginning of testing and kept mutually exclusive throughout testing. Participants were maintained at the University of North Dakota animal research facilities under controlled conditions of photoperiod (12h light to 12h dark) and temperature (22±1 °C) with ad libitum access to standard food, water, and nesting material. Mice were housed together in groups of 2–4, with wildtype controls being housed individually in instances where fighting or minor injuries were observed. All procedures involving animals were reviewed and approved by the UND Institutional Animal Care and Use Committee (IACUC) in accordance with the NIH guidelines for the care and use of laboratory animals.

### Fitness testing paradigm

Fitness tests were derived from the comprehensive functional assessment battery (CFAB), a set of noninvasive tests used to assess and compare the physical function and motor task ability of rodents [18]. All participants underwent fitness testing once per month over a period of 6 months. Grip strength and rotarod tests were performed on the same day, with endurance running testing taking place the following day to avoid fatigue/stress. Grip strength testing is used in the assessment of neuromuscular function and muscular strength by measuring the peak force required to make the subject release its grip. Rotarod is used as a performance and behavioral task to assess motor performance and walking gait by using the natural fear of falling as motivation [18]. Treadmill running was administered to measure the endurance capacity of the animals and the effects of aging on muscular endurance. The same researcher administered physical fitness tests to eliminate testing bias.

### All limb grip strength

The conventional forelimb grip strength test is a method to assess skeletal muscle function in rodents; in this study, we modified this method to include all four limbs to reduce variability and improve consistency. Grip strength was recorded via Maze Engineers Grip Strength Instrument (Skokie, IL, USA). The apparatus has a maximum tensile force range from 0 to 50 N, a reading accuracy of 0.1 g steps, and an error of ≤ 0.3 g. To measure grip strength, mice were gently lifted by the tail so that both the fore and hind paws could grasp the steel grid. Mice were then gently pulled away from the apparatus by the tail until the grip was released. Peak force was the objective of the assessment. On testing days, animals were first weighed and then given a set of 5 grip strength trials. For data normalization, the top and bottom scores from each animal were eliminated, with the remaining 3 trials being averaged and then divided by the animal’s bodyweight, in grams, to produce a final evaluation of relative fitness expressed in peak force/grams bodyweight.

### Rotarod

Rotarod testing was recorded utilizing Maze Engineers Rotarod Instrument (Skokie, IL, USA). The apparatus features dividers to prevent mice from entering other lanes and was further fitted with 50% smaller rotating dowels in half the lanes to accommodate the diminutive stature of dwarf mice. The rotarod dowels rotate at an increasing speed with mice attempting to remain on it rather than fall onto a platform just below. Testing consisted of 5 min maximum latency with rotation starting at 4 revolutions/min and increasing steadily at 7.2 revolutions/min^2^ until a maximum speed of 40 revolutions/min was reached. When mice fell from the rotating dowel, their latency was automatically recorded via the motion sensor on the apparatus. On testing days, mice performed 3 trials of rotarod with a break of>10 min between trials. Final scores were obtained by averaging the latency of three trials expressed in seconds.

### Endurance running

Endurance running testing was recorded via Maze Engineers Treadmill Instrument (Skokie, IL, USA). The treadmill features a single belt with 5 divided lanes and programmable speed/acceleration parameters. The treadmill was set at 0° incline and programmed to run for a maximum of 60 min with an acceleration rate of 1 meter/min^2^. On testing days, mice performed the test once. Data is expressed as absolute running time and then separately as a relative measure of total strides to exhaustion to normalize for the diminutive stride length of Ames dwarf mice.

### Stride length measurement

Male Ames dwarf and wildtype controls from middle-aged (8–12 months) and aged (18+ m.o.) had paws dipped in water-soluble paint and were placed on a large sheet of white paper to measure gait/stride length. Measurements were taken from the center of the right hind paw to the same hind paw center a stride later. Measurements were expressed in centimeters and revealed a comparative diminution in stride length of 25% (4.5 to 6 cm) for Ames dwarf mice.

### Tissue extraction

Following euthanasia with CO2 inhalation, tibialis anterior and quadratus femoris muscles were excised from both hindlimbs, with one mounted in OCT mounding media and rapidly frozen in liquid nitrogen-chilled isopentane for subsequent cryosectioning and histological analysis, and the other snap-frozen in liquid nitrogen. Lower leg mixed muscle groups (gastrocnemius, soleus, extensor digitorum longus) were then excised and frozen for future analysis.

### Tissue sectioning

Ten-micrometer-thick slices were sectioned from OCT-embedded tibialis anterior muscles beginning at the muscle midbelly at −25 °C using a Leica CM3050S Cryostat (Leica Biosystems; Deer Park, IL, USA). Slides were stored at −80 °C until analyzed for morphological characteristics.

### Laminin staining

Unfixed skeletal muscle (tibialis anterior) tissues were thawed briefly at room temperature and fixed with 4% paraformaldehyde for 10 min. Slides were permeabilized with 0.2% buffer of Triton-X in phosphate buffer saline (PBS) and then blocked with 10% goat serum solution prior to antibody incubation. Slides were then incubated with primary antibody against laminin (1:500, Abcam AB11575) for 12–18 h at 4 °C. Slides were washed with PBS three times and blocked again with 10% goat serum before incubation with secondary Alexa Fluor 546 goat anti-rabbit IgG (1:2000, Invitrogen A11035) biotinylated antibodies. Slides were then mounted with Permount (Thermo Fisher Scientific), sealed with coverslips, and imaged with EVOS (Thermo Fisher Scientific) fluorescence imaging system at 10× objective [19, 20].

### Hematoxylin and eosin (H&E) staining

On the day of staining, slides were brought to room temperature and loaded into the Autostainer XL (Leica ST5010) for automated staining transfers. Hematoxylin and eosin staining protocol is commonly used in the study of muscle development, growth, and regeneration as described [21].

### Picrosirius red staining

Staining for collagen fiber content was performed using a Picro-Sirius Red Stain Kit from STAT Lab (McKinney TX, USA). Slides were brought to room temperature, fixed with zinc formalin for 30 min to prevent tissue runoff, and stained with Weigerts Hematoxylin and Picro-Sirius Red Stain according to kit [19].

### MHC (myosin heavy chain) fiber-type staining

Tissue sections were thawed for 10 min at room temperature before being blocked with 10% goat serum in PBS for 1 h. Sections were then incubated with primary antibodies for 2 h at room temperature. Primary antibody conditions included anti-type I MHC IgG_2b_ (1:50) Developmental Studies Hybridoma Bank (#BA-F8), anti-type IIA MHC IgG_1_ (1:600) Developmental Studies Hybridoma Bank (#SC-71), and anti-type IIB MHC IgM (1:100) Developmental Studies Hybridoma Bank (#BF-F3), all prepared in 10% goat serum in PBS. Slides were then washed with PBS three times for 5 min each before being incubated with secondary biotinylated antibodies for 1 h at room temperature. Secondary antibody conditions included Alexa 350 goat anti-rabbit IgG_2b_ (1:500) (Invitrogen #A21140), Alexa 488 goat anti-mouse IgG_1_ (1:500) (Invitrogen #A21121), and Alexa 555 goat anti-mouse IgGM (1:500) (Invitrogen #A21426) prepared in 10% goat serum in PBS. Following secondary antibody incubation, slides were washed with PBS three times for 5 min each before being cover-slipped with ProLong Gold Antifade mounting medium. Imaging was performed with Leica DMi8 Thunder Imager at an objective of 10x.

### CSA analysis and quantification

Laminin-stained muscle sections were imaged at 10× with an EVOS (Thermo Fisher Scientific) microscope and then uploaded to ImageJ (Downloadable at ImageJ.com, produced at NIH, Bethesda, MD, USA). Using ImageJ, myofibers were analyzed with a CSA macro tool that segments and lists individual myofibers on an ROI manager, with listed measurements of each fiber’s area achieved at the end in an ROI measurement list. Fibers that were incorrectly segmented/measured by ImageJ macro were edited and fixed by hand by the researcher. A minimum of 250 myofibers was measured for each individual, and results were expressed as average myofiber CSA, expressed in microns^2^. This method is commonly described throughout the literature [19, 20].

### Fibrosis quantification

Picrosirius red-stained muscle sections were scanned and uploaded to ImageJ. Analysis was performed according to examples provided on the ImageJ website (https://imagej.net/ij/docs/examples/stained-sections/index.html).

### MHC fiber-type quantification

MHC-stained muscle sections were scanned and uploaded to ImageJ. Analysis was performed by doing a total count of fibers positive for MHC type 2A antibody. Data was expressed in both absolute counts of type 2A myofibers and as relative percentages by dividing MHC type 2A positive fibers by the total number of myofibers present. Relative measures were included to account for differences in total myofiber count between genotypes.

### Statistical analysis

Statistical analysis was performed by GraphPad Prism10.3.1 Software, (San Diego, CA, USA) using Two-way ANOVA followed by Tukey’s range test to quantify multiple comparisons with an alpha set of *p* <0.05. Group data is presented graphically as means ± standard error mean (SEM). Genotype, timepoint, and ages were used as variables.

## Results

### Dwarf mice are phenotypically diminutive compared to controls, reaching physical maturity later in life

**Fig. 1:**
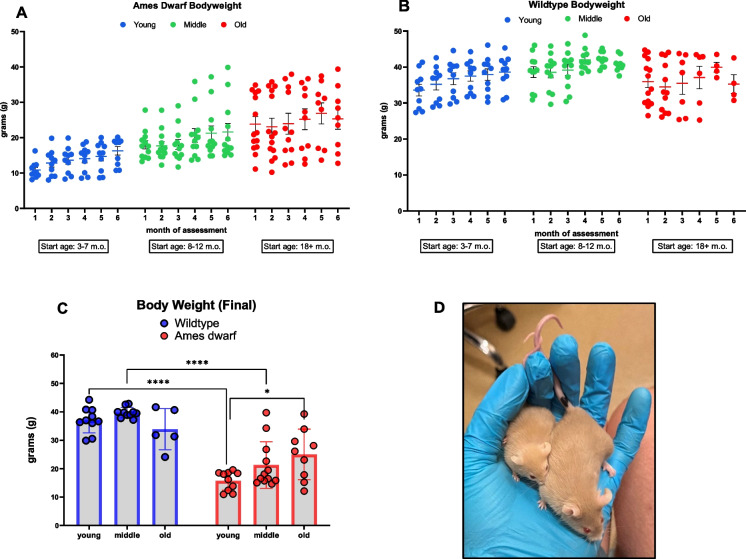
**Ames dwarf mice are diminutive relative to controls**. **A** Bodyweight of Ames dwarf mice across three age groups (young 3–7 months old, middle-aged 8–12 months old, and aged 18+ months old) recorded over 6 months, each recorded once/month prior to fitness testing (*N* = 31–35). Data expressed as means ± SEM. **B** Bodyweight of wildtype control mice across three age groups (young 3–7 months old, middle-aged 8–12 months old, and aged 18+ months old) recorded over 6 months, each recorded once/month prior to fitness testing (*N* = 24–34). Data expressed as means ± SEM. **C** Final postmortem bodyweight recorded directly after euthanasia (*N* = 25 for wildtype) (*N* = 31 for Ames dwarf). **D** Six-month old Ames dwarf (left) and wildtype (right) littermate

**Fig. 2:**
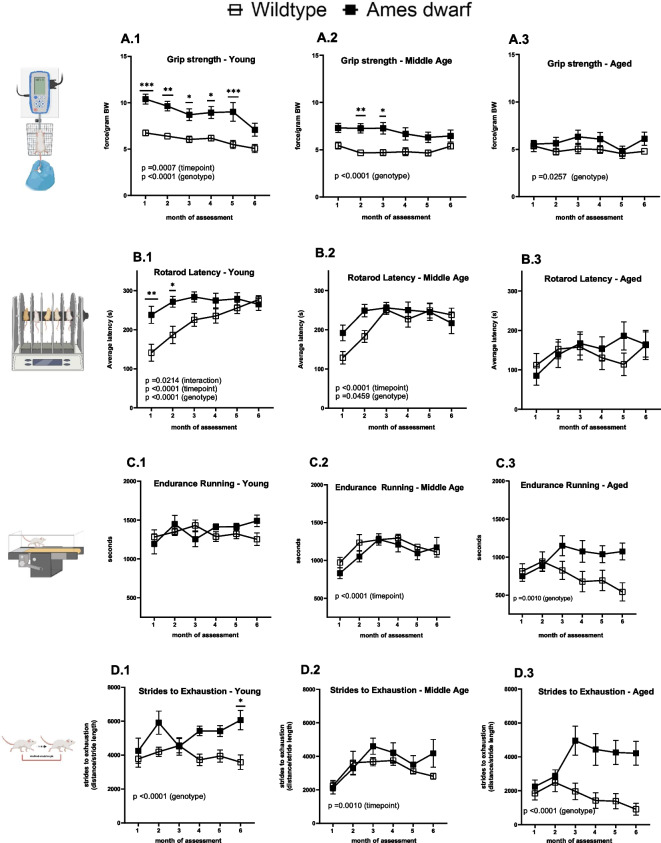
**Ames dwarf mice show no impairment in physical fitness, scoring equal to or higher than wildtype controls**.** A** Grip strength scores of young (3–7 months) dwarf (*N* = 10) and wildtype mice (*N* = 10), data expressed as mean +/− SEM. **A.2** Grip strength scores of middle-aged (8–12 months) dwarf (*N* = 12) and wildtype mice (*N* = 11). **A.3** Grip strength scores of aged (18+ months) dwarf (*N* = 9–12) and wildtype mice (*N* = 48). **B** Rotarod latency of young (3–7 months) dwarf (*N* = 10) and wildtype mice (*N* = 10), data expressed as mean +/− SEM. **B.2** Rotarod latency of middle-aged (8–12 months) dwarf (*N* = 12) and wildtype mice (*N* = 11). **B.3** Rotarod latency of aged (18+ months) dwarf (*N* = 9–12) and wildtype mice (*N* = 4–8). **C** Endurance running latency of young (3–7 months) dwarf (*N* = 10) and wildtype mice (*N* = 10), data expressed as mean +/− SEM. **C.2** Endurance running latency of middle-aged (8–12 months) dwarf (*N* = 12) and wildtype mice (*N* = 11). **C.3** Endurance running latency of aged (18+ months) dwarf (*N* = 9–11) and wildtype mice (*N* = 4–7). **D** Strides to exhaustion (total distance/stride length) of young (3–7 months) dwarf (*N* = 10) and wildtype mice (*N* = 10), data expressed as mean +/− SEM. **D.2** Strides to exhaustion (total distance/stride length) of middle-aged (8–12 months) dwarf (*N* = 12) and wildtype mice (*N* = 11). **D.3** Strides to exhaustion (total distance/stride length) of aged (18+ months) dwarf (*N* = 9–11) and wildtype mice (*N* = 4–7)

Ames dwarf mice in this study were approximately 40–60% smaller than wildtype control counterparts of the same age in young and middle-aged cohorts yet increased in bodyweight later into life (Fig. 1A, B). Wildtype controls reached physical maturity earlier in life (physical maturity characterized here as plateauing in body mass) around 8–10 months of age, with a moderate decline in weight observed in our aged group. The average body mass of Ames mice increased at a linear rate until mice were around 18 months old. To this point, the diminutive body mass of Ames dwarf mice in the young cohort partially contributed to high relative grip strength scores listed further below (Fig. 2 (A.1)). A noticeable variation in bodyweight was apparent in the aged Ames dwarf cohort, with multiple individuals exceeding 30 g of weight. These outlier “obese” dwarf mice had an excess accumulation of visceral adipose tissue and tested poorly, negatively impacting physical test scores.

### GH deficiency does not impair the relative fitness of Ames dwarf mice

To determine if the physical fitness of Ames dwarf mice is impaired due to GH deficiency, we administered three fitness tests derived from the comprehensive functional assessment battery (CFAB) [18]. CFAB is an established set of noninvasive tests used to assess and compare the physical function and motor task ability of rodents [18]. Previous reports on the musculature of Snell dwarf mice (phenotypically identical to Ames dwarf with synonymous hormonal deficiency) conclude that Snell dwarf mice have lower muscle mass and impaired physical function [22]. However, the physical fitness of Ames dwarf mice has yet to be thoroughly measured using CFAB-derived tests across age groups with sufficient “N.” Therefore, all limb grip strength, rotarod, and treadmill running tests were administered to mutually exclusive, chronologically aged-matched young, middle-aged, and aged cohorts of Ames dwarf mice and wildtype controls over 6 months. Performance scores derived from both genotypes at young and middle ages not only characterize developmental differences in physical performance between genotypes but also provide a point of reference to then gauge the consequences of aging on the physical performance scores of aged individuals. To this point, significant declines in rotarod and endurance running performance (but not grip strength) were observed beginning at 21 months in wildtype controls (Fig. 2 (B.3, C.3, D.3)), highlighting the consequences of sarcopenia on neuromuscular coordination and endurance running capacity.

### All limb grip strength—relative strength

Grip strength scores were expressed as force/bodyweight (grams), representing relative strength. This was done to account for the diminutive body mass of Ames dwarf mice being roughly 50% that of age-matched wildtype controls. Young and middle-aged Ames dwarf mice display significant (*p* < 0.0001) improvements in relative grip strength scores compared to age-matched wildtype controls (Fig. 2 (A.1, A.2)). Similarly, aged (18–24 m.o.) Ames dwarf mice maintained equivalent or improved relative grip strength scores (*p* = 0.0257) compared to aged wildtype controls, which is important as differences in body mass between genotypes are lesser at this age (Fig. 2 (A.3)). Grip strength scores of wildtype controls remained consistent throughout age groups (Fig. 2 (A.1–3)). Our data suggests that the relative grip strength of Ames dwarf mice is not impaired but rather superior to wildtype controls.

### Rotarod—neuromuscular coordination

Parameters of the rotarod test include a consistent increase in revolutions per minute before reaching a maximum latency of 300 s. Ames dwarf mice were placed on dowels scaled 50% smaller in diameter than control dowels to accommodate for size discrepancies between genotypes. The foreign instrumentation of the rotarod apparatus reflects an initial “learning curve” observable across age groups, as mice acclimate to balancing on the rotating dowel. Ames mice exhibited initial improvements in rotarod latency, reaching baseline performance much earlier within the 6-month testing period compared to wildtype controls. Apart from initial improvements, significant improvements were apparent for both genotype and timepoint analysis in the young and middle-aged cohorts (*p* < 0.0001; Fig. 2 (B.1, B.2)). Middle-aged dwarf mice also outperformed controls comprehensively (*p* = 0.0459; Fig. 2 (B.2)). Aged dwarf mice showed no improvements nor deficiency in rotarod performance compared to controls, yet scored lower than their younger counterparts, as did wildtype controls (Fig. 2 (B.3)). Rotarod performance of wildtype controls decreased substantially (≈40%) from middle-aged to aged groups (Fig. 2 (B.2, B3)). Aged dwarf mice performing poorly relative to their younger counterparts can be partially accredited to their increased visceral adiposity, as bodyweight is correlated with rotarod performance until mice get too fat.

### Endurance running capacity

Endurance capacity is an important functional assessment for both rodents and humans, utilized as a readout of physical well-being and a predictor of susceptibility to disease or death. With data expressed as absolute latency, Ames dwarf mice in young and middle-aged groups scored equal to or marginally better than wildtype controls (Fig. 2 (C.1, C.2)). Significant improvements became apparent within the aged cohort (*p* = 0.0010; Fig. 2 (C.3)). Aged dwarf mice consistently outperformed their aged normal counterparts beginning in the 21^st^ month of life. This timepoint (21 m.o.) marked a significant decrease in the endurance capacity of wildtype controls, where latency scores continually declined below 50% of younger wildtype cohorts (Fig. 2 (C.2, C.3)). Furthermore, when endurance scores are normalized to “strides to exhaustion” to account for the diminutive stride length of Ames dwarf mice, differences are further exacerbated across age groups in favor of Ames mice (*p* < 0.0001; Fig. 2 (D.1–3)). This measurement offers relative differences whereas absolute latency reflects absolute performance. Our data reflects both absolute and relative improved endurance running capacity of Ames mice.

### Dwarf mouse TA muscles and myofibers are diminutive compared to controls and resist age-related decline in CSA

**Fig. 3:**
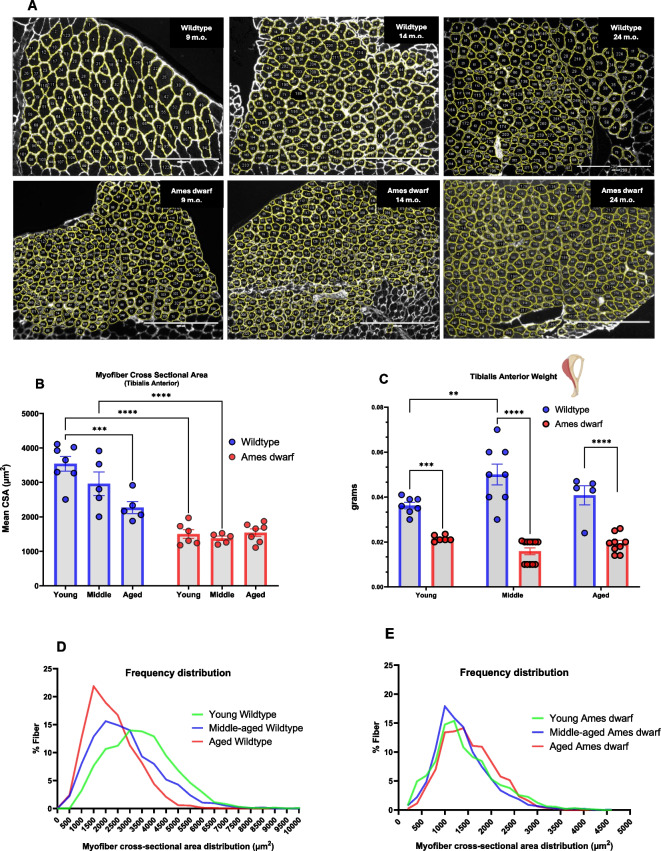

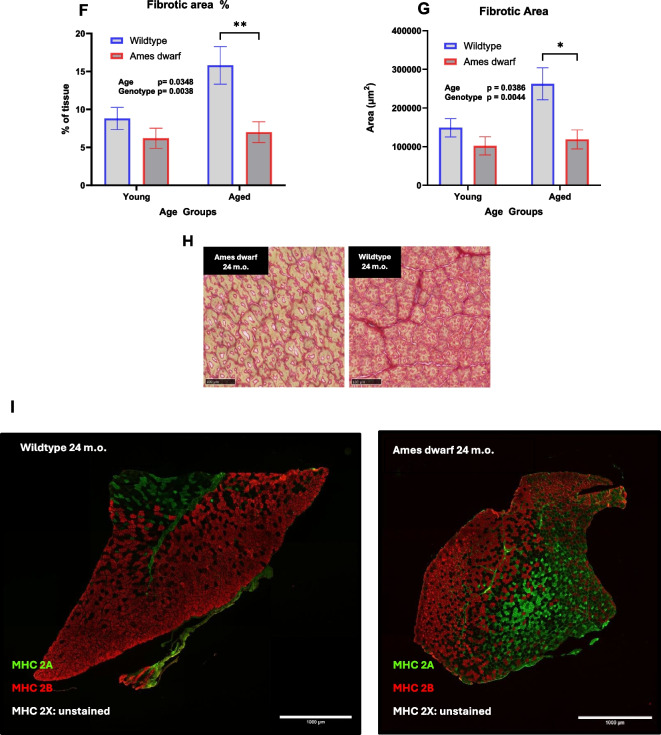

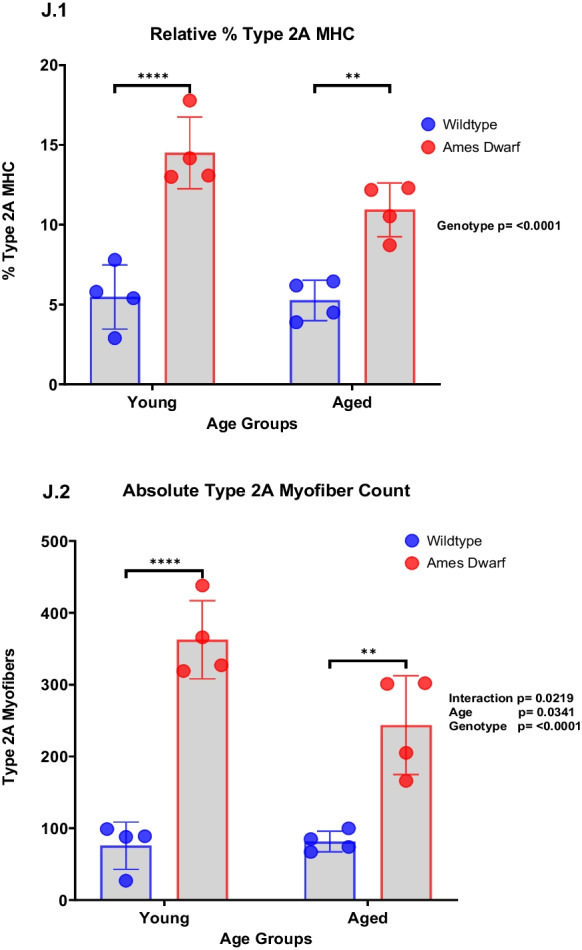
**Ames dwarf mice display diminutive myofiber cross-sectional area (CSA) relative to wildtype controls yet resist age-related decline in myofiber area and increases in fibrotic tissue**. **A** Laminin-stained skeletal muscle cross sections imaged at 10× with EVOS microscope, imported into ImageJ software, scale bar 400 µm. Individual myofibers outlined in yellow are recorded on an ROI manager that measures individual areas. **B** Average myofiber CSA of Ames dwarf mice and wildtype controls across age groups. Each data point represents a grouped average from one analyzed mouse, with a minimum of 250 myofibers analyzed/individual (*N* = 7 young wildtype, *N* = 6 young Ames dwarf, *N* = 5 middle-aged wildtype, *N* = 5 middle-aged Ames dwarf, *N* = 5 aged wildtype, *N* = 7 aged Ames dwarf). Fibers were analyzed using ImageJ software paired with a CSA macro tool. When necessary, cross-sectional analysis was performed on multiple images of Wildtype mice to obtain an adequate number of myofibers. Data is expressed as mean ± SEM with differences determined using two-ANOVA and Tukey’s multiple comparisons test. **C** Tibialis anterior weights for wildtype controls (*N* = 20) and dwarf mice (*N* = 27) across age groups. *N* values are low due to the earliest euthanized animals not having TA muscles weighed. Individual values were produced by averaging the weight of both left and right leg TA muscles from each mouse. **D** Frequency distribution plot of individual myofiber CSA distributions across age groups of wildtype controls. **E** Frequency distribution plot of individual myofiber CSA distributions across age groups of Ames dwarf mice. **F** Percentage of muscle cross-section that was determined to be fibrotic across young (*N* = 5 wildtype, *N* = 5 Ames dwarf) and aged (*N* = 5 wildtype, *N* = 6 Ames dwarf) animals. Images were analyzed with ImageJ. **G** Area of muscle cross-section determined to be fibrotic across young (*N* = 5 wildtype, *N* = 5 Ames dwarf) and aged (*N* = 5 wildtype, *N* = 6 Ames dwarf) animals. **H** Representative picrosirius-stained muscle sections of 24 months. Ames dwarf (left) and 24 months wildtype control (right). **I** MHC-stained cross sections of tibialis anterior muscle from 24 months wildtype control (left) and 24 months Ames dwarf (right). **J** Relative percentage and absolute counts of type 2A myofibers present in young (*N* = 4 wildtype, *N* = 4 Ames dwarf) and aged (*N* = 4 wildtype, *N* = 4 Ames dwarf) animals. Images were analyzed using ImageJ software

Morphological differences present in the tibialis anterior (TA) muscle of Ames dwarf mice included a significant (*p* < 0.0001) diminution in mean myofiber CSA compared to wildtype controls across both young and middle-aged cohorts (Fig. 3B). CSA derived from the aged Ames dwarf cohort again showed a noticeable although not statistically significant (*p* = 0.0957) difference relative to wildtype controls*.* Furthermore, aged wildtype mice showed a significant decrease (*p* = 0.0006) in myofiber CSA from that of young wildtype controls, pointing to the deleterious process of skeletal muscle aging where myofibers diminish in size/area. Importantly, this decrease in myofiber CSA was not apparent between the young and aged cohorts of Ames dwarf mice, suggesting maintenance of “youthful” myofiber size into later life. The age-related atrophy of myofiber CSA has been previously reported in TA muscles of house mice (*Mus musculus*), the equivalent of our wildtype controls, aligning with our observations between young and aged wildtype control cohorts [20]. To this point, Ames dwarfs express a higher number of myofibers per area unit of muscle (Supplemental data), although whole muscle size remains relatively smaller than wildtype controls (Fig. 3C).

Frequency plots representing myofiber subpopulation size distribution show an observable decrease in size (shift to the left on the plot) for aged wildtype mice compared to young and middle-aged cohorts (Fig. 3D), indicative that wildtype controls are subject to age-related myofiber atrophy at the 24-month mark. We postulate from the literature that observed myofiber atrophy is preferential towards large, glycolytic type 2B and 2X myofibers. Comparatively, myofiber CSA distribution plots of Ames dwarf mice show close resemblance in size distribution throughout age groups, indicating attenuation or complete absence of myofiber atrophy in Ames dwarf mice up to the 24-month mark (Fig. 3E). Of note, aged (24 months) Ames dwarf mice express a higher subpopulation of fibers with a CSA greater than 1500 µm^2^ than younger dwarf cohorts, suggesting continued myofiber growth rather than degeneration into later life (Fig. 3E).

### Ames dwarf mice resist muscular fibrosis

Analysis of muscle cross sections stained with picrosirius red—a dye that binds to collagen fibrils—suggests that Ames dwarf mice resist age-related increases in muscular fibrosis. Skeletal muscle fibrosis results from excess extracellular matrix (ECM) proteins, such as collagen, in the muscle. Fibrotic muscle suffers impairments in both contractility and regenerative potential. We observed significant differences (*p* = 0.0038) between genotypes for levels of fibrosis between young and aged individuals, with wildtype controls exhibiting a higher percentage of collagen/fibrotic tissue surrounding and infiltrating myofibers within the TA muscle (Fig. 3F–H). Aged wildtype controls being subject to increased fibrotic infiltration correlate to muscular impairment and may contribute to the decreased fitness performance of these individuals beginning at the 21-month mark.

### Ames dwarf mice express a higher percentage of oxidative type 2A muscle fibers, owing to muscular endurance

Analysis of MHC isoforms present in the tibialis anterior muscle reveals a significantly higher percentage of type 2A fast twitch fibers in Ames dwarf mice compared to controls in both young and aged cohorts (*p* = <0.0001; Fig. 3I–J). Significant differences were observed when expressing data in both relative percentage of MHC type 2A frequency and in absolute counts of MHC type 2A fibers (Fig. 3 (J.1, J.2)). Type 2A myofibers are relatively rich in mitochondria, particularly when compared to other fast-twitch muscle fiber types like 2X and 2B, making them more oxidative and capable of sustained activity compared to purely glycolytic fibers, which are characterized by their short termed, high force producing capacity. Of note, the tibialis anterior muscle is almost entirely composed of fast twitch fibers, and the increased oxidative potential in a fast twitch muscle group directly correlates with the improved endurance running capacity of Ames dwarf mice. Differences across age groups included a moderate decline of MHC type 2A frequency in both genotypes, although wildtype controls remained relatively stable, pointing to complete isoform evolution towards large glycolytic myofibers in normal mice.

## Discussion

Our chief findings regarding the skeletal muscle of the Ames dwarf mouse include enhanced endurance running capacity relative to wildtype controls—particularly in mice older than 18 months, paired with attenuation of sarcopenic processes of myofiber atrophy, fiber type, and fibrotic infiltration. Elevated frequency of oxidative, type 2A myofibers in aged Ames mice provides a metabolic benefit to muscular endurance and resistance to sarcopenia. In contrast, declines in both myofiber integrity and fitness performance were observed in wildtype controls, signaling that wildtype mice are subject to the deleterious process of aging on muscle health before 24 months of age. Considering that skeletal muscle is increasingly implicated as a key regulator of aging and longevity [17, 18, 23–26], our study was designed to characterize age-related differences in fitness performance and muscle morphology between an established longevity model in the Ames dwarf mice and wildtype controls. Despite multiple longevity studies utilizing various dwarf mouse strains, comprehensive direct measures of the physical performance of the Ames dwarf strain remain limited. While previous studies correlated Ames dwarfism with improved maintenance of neuromusculoskeletal tasks into old age [17], the Snell dwarf mouse, a phenotypic analog, is continually regarded as physically impaired with weak muscles [22]. Given the absence of GH and very low levels of IGF-1, it could be expected that both muscle size and function of Ames dwarf mice would be impaired. Despite this, our findings support the paradoxical relationship of GH-deficient mice exhibiting improved muscle health and function into old age. Furthermore, morphological differences present in Ames dwarf skeletal muscle suggest cellular protection against age-related myofiber atrophy and infiltration of fibrotic proteins, paired with improved oxidative capacity and fatigue resistance relative to controls.

### Fitness tests as readout of physical capacity

We derived three fitness tests (grip strength, rotarod, treadmill) from the comprehensive functional assessment battery (CFAB) [18] to compare physical performance trends between genotypes and age groups. Importantly, execution of these physical tests demands coordination of multiple organ systems (nervous, muscular, and skeletal), thereby offering a more comprehensive read-out of individual physiological status rather than exclusively assessing muscle tissue in an in vitro context. Accommodations were made in either instrument orientation or data normalization to accommodate for the 50% size diminution of the Ames dwarf. To this point, the grip strength testing grid was angled more horizontally to allow mice to pull with all four limbs, contrary to other studies limiting pulling force to the forelimbs only. Grip strength scores were also expressed relative to bodyweight (grams), a step we deemed practical and necessary in accounting for the somatic growth retardation of dwarf mice. Normalizing grip strength scores of young Ames dwarf mice to their very low bodyweights at this age (10–20 g) can be partially accredited for relative improvements noticed at this age, yet provides valuable insight nonetheless. For rotarod testing, speed and time parameters remained constant while the dowels that dwarf mice were placed on were sized down 50% to accommodate for size discrepancies, thereby providing a relatively equal surface for both genotypes to balance on. Conditions for treadmill endurance running testing remained constant between genotypes with normalization applied in additional data presentation as strides to exhaustion to account for the diminutive stride length of Ames dwarf mice.

We designed our physical fitness assessment of the Ames dwarf with as much correlation to human clinical frailty assessment as possible. In both humans and mice, a decline in grip strength has been associated with increases in all-cause mortality and diseases such as diabetes, cardiovascular disease, and cancer [24, 27]. A valuable assessment of the general health of aging adults, grip strength acts as a predictor for future bone and muscle function as well as cognitive decline and dementia [24]. The rotarod test evaluates gait speed and balance, another clinical assessment commonly administered to older adults [18]. Endurance running, albeit more strenuous of a test, provides absolute values of physical performance and further elucidates any possible physical or cognitive deficiencies. We believe that administering tests that correlate to human frailty assessment to mutually exclusive age groups of mice over a testing period of 6 months provides true longitudinal quantification of the relative fitness capacity of GH-mutant Ames dwarf mice relative to wildtype controls. Our data suggests that the anti-aging effects of the Prop-1 mutation extend to skeletal muscle function and fatigue resistance—particularly endurance running capacity into old age.

We found that the physical performance of male Ames dwarf mice was equal to or superior to age-matched wildtype controls across three distinct measurements: grip strength, rotarod, and treadmill endurance running. Ames dwarf mice of young and middle-aged cohorts showed initial improvements in grip strength and rotarod capabilities compared to wildtype controls, with differences narrowing in aged cohorts. These mice also reached maximum rotarod latency (300s) much earlier in the 6-month testing process than wildtype controls, possibly pointing to improved cognitive performance of Ames dwarf mice. This is supported by previous work done using the Barnes maze and T maze tests suggesting improved cognitive function of Ames dwarf mice into old age [28, 29].

A major finding of our study was the improved endurance running capacity of Ames dwarf mice in the aged cohort, where they increasingly outperformed wildtype controls in both absolute running latency as well as in relative measures of strides to exhaustion. Improved capacity for low-moderate intensity exercise such as endurance running may be attributed to the increased ratio of oxidative to glycolytic myofibers in the Ames dwarf, a result of myofiber isoform evolution retardation [30]. The increased frequency of oxidative myofibers in the tibialis anterior of the Ames dwarf provides a metabolic mechanism to support our findings.

While significance favoring the Ames dwarf was not detected in rotarod or grip strength scores between aged cohorts, the “absence of impairment” or relative trend of performance observed helps negate the idea that GH-deficient mice exhibit innate physical fitness deficiencies. Importantly, an observable decline in rotarod and endurance running capacity was observed in wildtype controls starting at 21 months of age, highlighting the deterioration of neuromuscular performance that is otherwise absent in GH-free Ames mice.

### Skeletal muscle morphology of Ames dwarf mouse

We confirmed the mean myofiber CSA of Ames dwarf mice to be comparatively diminutive to that of wildtype controls, which was expected due to somatic growth retardation characteristic of GH-deficiency. Apart from size differences, muscle from Ames dwarf mice expresses more myofibers per area, with H&E staining revealing healthy nucleation, proper fascicular organization, and cuboidal-shaped myofibers forming tight junctions with each other (Supplemental data).

Our chief finding is that skeletal muscle from GH-deficient Ames dwarf mice displays attenuation of several hallmarks of sarcopenia observable throughout age groups of wildtype controls, particularly myofiber atrophy, fibrotic infiltration, and loss of performance. The maintenance of youthful myofiber size in aged Ames dwarf mice may correlate with relative improvements in physical fitness scores observable across the 18–24-month mark. Furthermore, frequency distribution data suggests that hormonally normal wildtype controls are subject to preferential atrophy of large, glycolytic myofibers, as CSA distribution plots reveal a noticeable loss of large (>2500 µm^2^) myofiber subpopulations in the aged cohort, whereas myofiber size frequency distribution plots of young and aged Ames dwarf mice show very close resemblance to one another**.**

When observing fibrosis levels within the muscle, our data suggests a significant increase in fibrotic tissue from young to aged wildtype controls, whereas Ames dwarf mice did not change with age. Importantly, fibrotic analysis was performed by imaging both genotypes at the same objective/magnification, thereby including a higher number of Ames dwarf myofibers per objective frame. To this point, we hypothesize that if fibrotic analysis was normalized to an identical ratio of muscle tissue between genotypes or was performed on whole muscle images, the differences would be further exacerbated in favor of the Ames dwarf. Skeletal muscle fibrosis is caused by the over-deposition of extracellular matrix (ECM) proteins, particularly collagen, and is strongly associated with inflammation, specifically TGF-β [31, 32]. While ECM has a principal role in muscle repair following injury, sarcopenic muscle is unable to perform the subsequent clearance of fibrotic protein once healed, creating a feedback loop of ECM deposition and increased muscular inflammation. Muscular fibrosis begins with continual thickening of the endomysium and progresses to eventual infiltration of collagen proteins into the myofibers themselves. Loss of contractile surface area negatively affects muscular strength and regenerative capacity [31]. We hypothesize that Ames dwarfs are more resistant to fibrotic infiltration than wildtype controls due to their lower levels of GH and thyroid hormone, which can otherwise promote fibrotic signaling in aging tissue. Previous studies have shown that GH-deficient dwarf mice generally exhibit less fibrosis in various organs like the heart, kidneys, and liver compared to their non-dwarf counterparts [32]. Furthermore, Ames mice may show attenuation of skeletal muscle fibrosis due to downregulated TGF-β pathway signaling and improved maintenance of skeletal muscle niche cell types (macrophage, FAP cells) that facilitate the proper clearance of fibrotic proteins [31]. Preliminary RNA-seq data derived from male Ames and wildtype mixed muscle shows increased expression of PGC-1α in male Ames dwarf mice (data not shown). PGC-1α is known to promote mitochondrial biogenesis, thereby reducing ROS and cellular stress.

Furthermore, differences in metabolic signatures of myofiber populations between genotypes seem to favor the Ames dwarf in terms of both resistance to sarcopenia and improved endurance capacity. We observed a significantly increased percentage of oxidative fast-twitch type 2A myofibers present in the tibialis anterior muscle of Ames mice. This altered MHC profile supports their enhanced endurance running capacity, as wildtype controls express an almost exclusive type 2B or 2X myofiber population profile, rendering them more susceptible to fatigue and the negative effects of sarcopenia, as large glycolytic type 2B and 2X fibers are the first to atrophy and become dysfunctional with age [25, 26]. Myosin heavy chain evolution has been previously shown to be retarded in both cardiac and skeletal muscle in other strains of hypopituitary dwarf mice, further supporting our findings [30]. Thus, avoiding the final evolution to full glycolytic type 2B or type 2X myofibers in a fast twitch muscle like the tibialis anterior seems to provide an innate metabolic advantage to the Ames dwarf in the context of endurance activities. Dwarf mice displayed a two-to-three-fold increase in the relative percentage of oxidative fast twitch type 2A myofibers, and we hypothesize that this difference might be further exacerbated in slow-twitch muscle groups that traditionally express more type 1 myofibers, such as the soleus - but is outside the scope of this particular study.

Overall, Ames dwarf mice resisting numerous aspects of skeletal muscle aging that are present in age-matched controls supports our initial postulations that the pro-longevity Prop1 mutation would extend its effects to skeletal muscle, providing further validity to the relationship between skeletal muscle maintenance and mammalian longevity.

### Skeletal muscle and aging

Skeletal muscle health and performance are increasingly implicated and validated as a predictor of susceptibility to disease and overall mortality in rodents and humans—justifying its significance in the context of longevity [24–26]. This relationship strengthens our idea that Ames dwarf mice benefit from their enhanced longevity at least in part due to improved maintenance of their largest organ system—skeletal muscle, specifically retention of youthful myofiber morphology and physical fitness capacity into old age.

Preservation of muscle mass and function into later life drastically reduces vulnerability to external stressors and the probability of developing multiple co-morbidities, possibly through improved cellular function, glucose storage, or positive paracrine influence on other organ systems. Functional skeletal muscle permits the performance of daily tasks, ambulation, proper posture, and respiratory mechanics and regulates whole-body metabolism [24, 27]. The age-related loss of skeletal muscle mass and strength is clinically defined as sarcopenia [23, 25, 26]. Sarcopenia is a natural, progressive aspect of aging that particularly accelerates in later life, posing an ominous issue to increasingly aged populations of Western nations. Lifestyle changes associated with increased age include reduced physical activity, poor diet, and metabolic dysregulation—all thought to contribute to sarcopenic progression. At the cellular level, loss of myofiber size, preferential atrophy of glycolytic fast-twitch (type 2B and 2X) myofibers, over-deposition of fibrotic tissue, reduced regenerative capacity, and dysfunctional innervation are all associated with the decline in musculoskeletal performance [23, 25]. In addition to muscular weakness, older adults also suffer from an earlier onset of muscle fatigue and inability to maintain balance, leading to increased incidence of falls and injury that negatively impact health and survival metrics. Without regular exercise, particularly resistance training as a mitigating step, progressive muscle loss and weakness contribute to frailty, reduced quality of life, and susceptibility to co-morbidities and disease. While the anabolic properties of GH/IGF-1 signaling absolutely extend to build skeletal muscle mass and bone density during development, their importance and circulating levels wane following sexual maturation and continually decline throughout adulthood [7–9]. Low levels of GH have been previously implicated as a causative factor contributing to sarcopenia; however, these claims remain largely unsupported or oversimplified at best. Addressing the role of GH signaling in the progressive decline of skeletal muscle function serves to benefit an increasing number of individuals who are otherwise posed to experience a stark reduction in healthspan in later life due to sarcopenia.

### GH and aging process

The idea that GH deficiency (GHD) is the primary factor behind the enhanced longevity of Ames dwarf mice is inversely supported in murine and human models. GH-transgenic mice and humans with gigantism are both characterized by excess GH production and consequently, increased disease rates, and shortened lifespans [2, 7–11]. Further investigations across multiple species continue to provide ample evidence that GH is directly implicated in longevity. Extension of lifespan is apparent in other dwarf mouse species with mutations disrupting either GH synthesis or the GH receptor itself (*Snell, Little, GHRKO*) [7, 9]. Snell dwarf mice share the same three hormonal deficiencies as Ames and a comparable extension of lifespan—with mutations on different loci [8, 9, 22]. Furthermore, survival studies performed in model organisms (yeast, worms, and drosophila) with mutated genes encoding insulin/IGF-1 homologs further confirm the link between insulin signaling and lifespan [9, 33]. Ames dwarfism can be viewed as a genetic exchange of cellular growth for cellular protection early in life, a characteristic that seems to be a fundamental property of many organisms throughout the aging process. From mice to dogs to humans, GH levels continually decline after reaching physical maturity, suggesting a shift from the promotion of cellular growth to protection, reducing replicatory mechanisms as cellular dysfunction becomes more probable with age [3, 7–9]. The gradual loss of somatic GH activity was initially viewed as a negative symptom of aging but instead can be viewed as part of a metabolic regulatory process shifting towards cellular maintenance over cellular proliferation and lipolysis. Ames dwarf mice exemplify this shift and support the antagonistic pleiotropy theory of aging, the notion that suggests certain genes/characteristics can have beneficial effects early in an organism’s life, but detrimental effects later in life [7, 9].

### Controversy of GH therapy in humans

Humans with a pituitary hormone deficiency, or growth hormone deficiency (GHD) are not gifted with the robust extension of longevity characteristic of their murine counterparts but do display innate resistance to diabetes and cancer [8]. In terms of skeletal muscle, both GHD humans and older adults with low plasma GH experience declines in muscle mass and strength paired with increased visceral adiposity [7, 8]. The anabolic and lipolytic activities of GH replacement therapy in those diagnosed with GHD help young individuals undergo proper prepubescent growth, or, in adults, help build muscle mass and reduce visceral adiposity—improving body composition and overall quality of life [7–9]. Initially, this spurred intense interest and confidence that recombinant human GH therapy could be an “anti-aging” agent of the future. However, further studies elucidated the negative side effects of GH therapy such as inflammation, edema, and autoimmune reactions [7, 8]. Furthermore, there is little evidence that administering GH alone enhances physical performance in healthy adults, as studies in hormonally normal adult men have shown that muscle strength, power, and aerobic exercise capacity are not improved by exogenous GH administration, with improvements reserved for those who exhibit clinical deficiency [2, 7, 8]. Administrating GH to hormonally normal individuals is particularly dangerous due both to its anti-insulinemic effects and the impact growth factors have on the increased incidence and progression of neoplasms [8]. The current consensus among researchers is that while GH deficiency is a valid indication for GH therapy, old age without diagnosable disruption of GH signaling is not. Rightfully so, prescribing GH to endocrinologically normal individuals under the intention of delaying or reversing aging is largely ineffective and more importantly illegal to practice in the USA [8].

### Limitations of study

All mice used in our study were male, thus rendering our findings sex-limited. Male and female Ames mice display differences in sex hormones, robustness of longevity, and fertility, whereas differences in body size and muscular performance are seemingly minor. Under normal circumstances, male rodents are often larger than females; however, testosterone levels of Ames dwarf mice are very low, rendering this difference negligible in our model. Female dwarf mice generally exhibit a more robust extension in lifespan, as the mean lifespan increase is 68% compared to 49% in males [34]. While both male and female Ames dwarf mice express impairments in fertility, females are unable to carry pregnancies due to a lack of prolactin, which is required for appropriate ovarian function in mice. For this reason, male homozygous Ames mice are bred with heterozygous female carriers that are phenotypically normal. As for muscle morphology and performance, no sex-specific differences have been elucidated. However, longitudinal studies are ongoing in our lab that incorporate female Ames dwarf and wildtype controls in fitness evaluation tests as well as investigating any potential morphological differences in cross-sectional area or myofiber integrity throughout the aging process. We suspect that differences in strength or fitness capacity between male and female Ames mice will be minor due to low plasma sex steroid levels. Male mice were chosen due to availability at the time of study commencement. Furthermore, the “N” of our aged cohorts were negatively affected by natural mortality, as initially each aged (18+ months) cohort numbered 10–12 individuals/genotype, with aged wildtype controls decreasing to 4–7 individuals by the end point of testing, when mice were 24 months old. This reduction in “N” limited statistical significance within the aged cohort.

## Conclusions

Our findings support the paradoxical relationship between GH-deficient Ames dwarf mice expressing relative and absolute improvements in certain physical fitness measures compared to wildtype controls. Myofiber analysis suggests that 24-month-old Ames dwarf mice resist sarcopenic processes of myofiber atrophy, fibrotic infiltration, and loss of performance. The enhanced endurance running capacity of Ames dwarf mice is in part attributable to increased oxidative processes within the muscle, as we found a two-to-three-fold increase in the relative percentage of oxidative type 2A myofibers present in tibialis anterior muscle compared to wildtype controls. Our study offers novel insight into how a longevity mutation extends to skeletal muscle, elucidating positive differences in both performance and morphology in the absence of postnatal somatotropic signaling.

## Supplementary Information

Below is the link to the electronic supplementary material.

Supplementary file1 (PDF 409 KB)

## References

[1] Brown-Borg HM, Borg KE, Meliska CJ, Bartke A. Dwarf mice and the ageing process. Nature. 1996;384(6604):33–33. 10.1038/384033a0. 8900272 10.1038/384033a0

[2] Bartke A, Brown-Borg H. Life extension in the dwarf mouse. In: Current Topics in Developmental Biology. Vol 63. Academic Press; 2004:189-225. 10.1016/S0070-2153(04)63006-7 10.1016/S0070-2153(04)63006-7 15536017

[3] Bartke A, Wright JC, Mattison JA, Ingram DK, Miller RA, Roth GS. Extending the lifespan of long-lived mice. Nature. 2001;414(6862):412–412. 10.1038/35106646. 11719795 10.1038/35106646

[4] Brown-Borg H, Johnson WT, Rakoczy S, Romanick M. Mitochondrial oxidant generation and oxidative damage in Ames dwarf and GH transgenic mice. J Am Aging Assoc. 2001;24(3):85–96. 10.1007/s11357-001-0012-6. 23604879 10.1007/s11357-001-0012-6 PMC3455482

[5] Brown-Borg HM. Hormonal regulation of longevity in mammals. Ageing Res Rev. 2007;6(1):28–45. 10.1016/j.arr.2007.02.005. 17360245 10.1016/j.arr.2007.02.005 PMC1978093

[6] Brown-Borg HM, Rakoczy SG. Growth hormone administration to long-living dwarf mice alters multiple components of the antioxidative defense system. Mech Ageing Dev. 2003;124(10–12):1013–24. 10.1016/j.mad.2003.07.001. 14659590 10.1016/j.mad.2003.07.001

[7] Bartke A. Somatotropic axis, pace of life and aging. Front Endocrinol (Lausanne). 2022;13:916139. 10.3389/fendo.2022.916139. 35909509 10.3389/fendo.2022.916139 PMC9329927

[8] Bartke A. Growth hormone and aging: updated review. World J Mens Health. 2018;37(1):19. 10.5534/wjmh.180018. 29756419 10.5534/wjmh.180018 PMC6305861

[9] Bartke A, Brown-Borg HM, Bode AM, Carlson J, Hunter WS, Bronson RT. Does growth hormone prevent or accelerate aging? Exp Gerontol. 1998;33(7–8):675–87. 10.1016/S0531-5565(98)00032-1. 9951615 10.1016/s0531-5565(98)00032-1

[10] Bartke A, Brown-Borg H, Kinney B, et al. Growth hormone and aging. J Am Aging Assoc. 2000;23(4):219. 10.1007/s11357-000-0021-x. 23604867 10.1007/s11357-000-0021-x PMC3455269

[11] Bartke A, Hascup E, Hascup K, Masternak MM. Growth hormone and aging: new findings. World J Mens Health. 2021;39(3):454–65. 10.5534/wjmh.200201. 33663025 10.5534/wjmh.200201 PMC8255405

[12] Kennedy MA, Rakoczy SG, Brown-Borg HM. Long-living Ames dwarf mouse hepatocytes readily undergo apoptosis. Exp Gerontol. 2003;38(9):997–1008. 10.1016/s0531-5565(03)00164-5. 12954487 10.1016/s0531-5565(03)00164-5

[13] Brown-Borg HM, Rakoczy S, Wonderlich JA, Armstrong V, Rojanathammanee L. Altered dietary methionine differentially impacts glutathione and methionine metabolism in long-living growth hormone-deficient Ames dwarf and wild-type mice. Longev Healthspan. 2014;3(1):10. 10.1186/2046-2395-3-10. 25584190 10.1186/2046-2395-3-10 PMC4290132

[14] Romanick MA, Rakoczy SG, Brown-Borg HM. Long-lived Ames dwarf mouse exhibits increased antioxidant defense in skeletal muscle. Mech Ageing Dev. 2004;125(4):269–81. 10.1016/j.mad.2004.02.001. 15063102 10.1016/j.mad.2004.02.001

[15] Kim G, Kim JH. Impact of skeletal muscle mass on metabolic health. Endocrinol Metab (Seoul). 2020;35(1):1–6. 10.3803/EnM.2020.35.1.1. 32207258 10.3803/EnM.2020.35.1.1 PMC7090295

[16] Larsson L, Degens H, Li M, et al. Sarcopenia: aging-related loss of muscle mass and function. Physiol Rev. 2019;99(1):427–511. 10.1152/physrev.00061.2017. 30427277 10.1152/physrev.00061.2017 PMC6442923

[17] Arum O, Rasche ZA, Rickman DJ, Bartke A. Prevention of neuromusculoskeletal frailty in slow-aging ames dwarf mice: longitudinal investigation of interaction of longevity genes and caloric restriction. PLoS One. 2013;8(10):e72255. 10.1371/journal.pone.0072255. 24155868 10.1371/journal.pone.0072255 PMC3796515

[18] Graber T, Fry C, Marota R, Rasmussen B. CFAB: comprehensive functional assessment battery for older mice. Innovation in Aging. 2018; 2(suppl_1):879. 10.1093/geroni/igy031.3281

[19] Kiernan J. Histological and histochemical methods: theory and practice Vol 6th. Hodder Arnold Publications. 2015.

[20] Zhang FM, Wu HF, Wang KF, et al. Transcriptome profiling of fast/glycolytic and slow/oxidative muscle fibers in aging and obesity. Cell Death Dis. 2024;15(6):1–12. 10.1038/s41419-024-06851-y. 38942747 10.1038/s41419-024-06851-y PMC11213941

[21] Wang C, Yue F, Kuang S. Muscle histology characterization using H&E staining and muscle fiber type classification using immunofluorescence staining. Bio Protoc. 2017;7(10):e2279. 10.21769/BioProtoc.2279. 28752107 10.21769/BioProtoc.2279 PMC5526362

[22] Rader EP, McKinstry KA, Baker BA. Transcriptional and morphological responses following distinct muscle contraction protocols for Snell dwarf (Pit1dw/dw) mice. Physiological Rep. 2024;12(17):e70027. 10.14814/phy2.70027. 10.14814/phy2.70027 PMC11371489 39227324

[23] Ardeljan AD, Hurezeanu R. Sarcopenia. In: *StatPearls*. StatPearls Publishing; 2024. http://www.ncbi.nlm.nih.gov/books/NBK560813/. Accessed 25 Oct 25, 2024.

[24] Celis-Morales CA, Welsh P, Lyall DM, et al. Associations of grip strength with cardiovascular, respiratory, and cancer outcomes and all-cause mortality: prospective cohort study of half a million UK Biobank participants. BMJ. 2018;361:k1651. 10.1136/bmj.k1651. 29739772 10.1136/bmj.k1651 PMC5939721

[25] Delmonico MJ, Beck DT. The current understanding of sarcopenia: emerging tools and interventional possibilities. Am J Lifestyle Med. 2017;11(2):167–81. 10.1177/1559827615594343. 30202329 10.1177/1559827615594343 PMC6125026

[26] Evans WJ, Guralnik J, Cawthon P, et al. Sarcopenia: no consensus, no diagnostic criteria, and no approved indication—how did we get here? GeroScience. 2023;46(1):183. 10.1007/s11357-023-01016-9. 37996722 10.1007/s11357-023-01016-9 PMC10828356

[27] Sayer AA, Dennison EM, Syddall HE, Gilbody HJ, Phillips DIW, Cooper C. Type 2 diabetes, muscle strength, and impaired physical function: the tip of the iceberg? Diabetes Care. 2005;28(10):2541–2. 10.2337/diacare.28.10.2541. 16186295 10.2337/diacare.28.10.2541

[28] Sharma S, Haselton J, Rakoczy S, Branshaw S, Brown-Borg HM. Spatial memory is enhanced in long-living Ames dwarf mice and maintained following kainic acid induced neurodegeneration. Mech Ageing Dev. 2010;131(6):422–35. 10.1016/j.mad.2010.06.004. 20561541 10.1016/j.mad.2010.06.004 PMC4104773

[29] Sharma S, Rakoczy S, Brown-Borg H. Assessment of spatial memory in mice. Life Sci. 2010;87(17–18):521–36. 10.1016/j.lfs.2010.09.004. 20837032 10.1016/j.lfs.2010.09.004 PMC6457258

[30] Butler-Browne GS, Prulière G, Cambon N, Whalen RG. Influence of the dwarf mouse mutation on skeletal and cardiac myosin isoforms. Effect of one injection of thyroxine on skeletal and cardiac muscle phenotype. J Biol Chem. 1987;262(31):15188–93. 10.1016/S0021-9258(18)48156-2. 3667629

[31] Mahdy MAA. Skeletal muscle fibrosis: an overview. Cell Tissue Res. 2019;375(3):575–88. 10.1007/s00441-018-2955-2. 30421315 10.1007/s00441-018-2955-2

[32] Salmon AB, Murakami S, Bartke A, Kopchick J, Yasumura K, Miller RA. Fibroblast cell lines from young adult mice of long-lived mutant strains are resistant to multiple forms of stress. Am J Physiol Endocrinol Metab. 2005;289(1):E23-29. 10.1152/ajpendo.00575.2004. 15701676 10.1152/ajpendo.00575.2004

[33] Tatar M, Kopelman A, Epstein D, Tu MP, Yin CM, Garofalo RS. A mutant Drosophila insulin receptor homolog that extends life-span and impairs neuroendocrine function. Science. 2001;292(5514):107–10. 10.1126/science.1057987. 11292875 10.1126/science.1057987

[34] Amador-Noguez D, Zimmerman J, Venable S, Darlington G. Gender-specific alterations in gene expression and loss of liver sexual dimorphism in the long-lived Ames dwarf mice. Biochem Biophys Res Commun. 2005;332(4):1086–100. 10.1016/j.bbrc.2005.05.063. 15925325 10.1016/j.bbrc.2005.05.063

