# Supplementary material for: Growth hormone–deficient Ames dwarf mice resist sarcopenia and exhibit enhanced endurance running performance at 24 months
Source: GeroScience. 2025 Mar 26;47(3):4827–43. doi: 10.1007/s11357-025-01630-9 (PMC12181600; doi:10.1007/s11357-025-01630-9)
Supplement: Supplementary file 1 — Supplementary file1 (PDF 409 KB) [file 11357_2025_1630_MOESM1_ESM.pdf]

Supplemental Data

Fig. 1

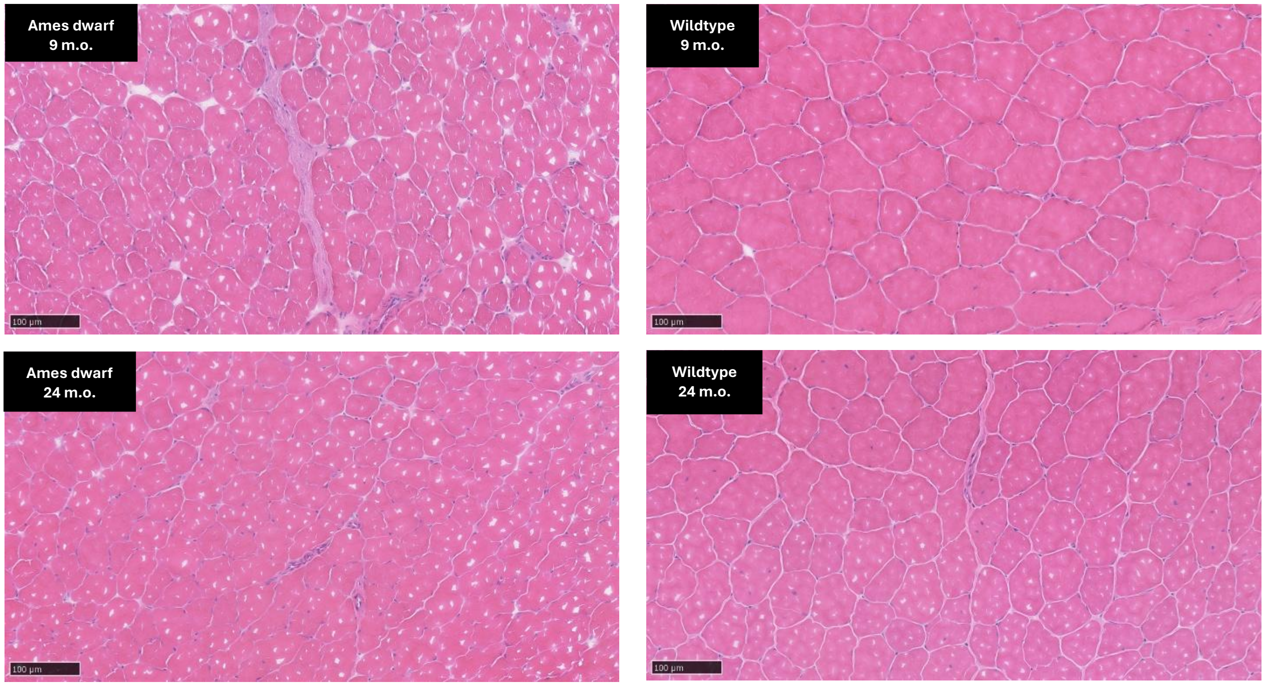

Supplemental Figure 1

Ames dwarf mice display healthy nucleation and fascicular organization. Hematoxylin and eosin staining of skeletal muscle tissue from Ames dwarf (left) and wildtype controls (right). 20x images were taken from young (top) and aged (bottom) mice. Myofibers are stained pink with nuclei stained blue.

Fig. 2

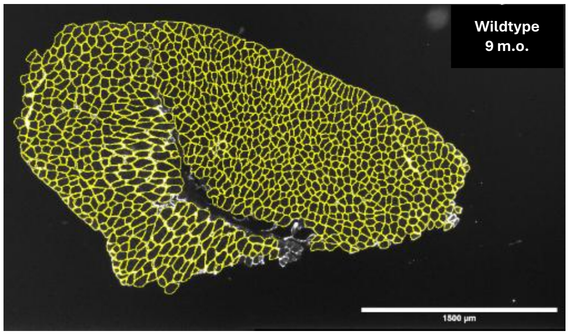

Wildtype control

Total myofibers: 1102

Mean CSA: 3697.99 µm

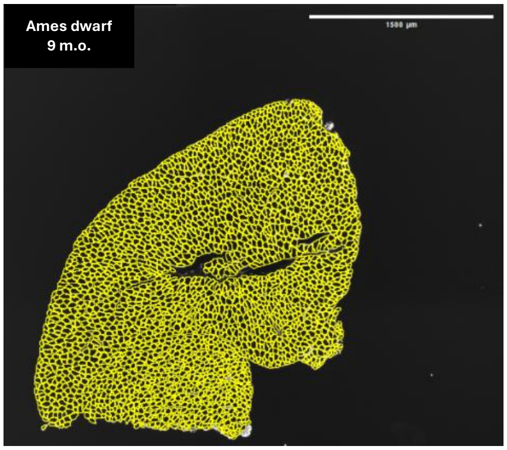

Ames dwarf

Total myofibers: 3452

Mean CSA: 1094.89µm

Supplemental Figure 2

Ames dwarf mice display more myofibers per muscle than wildtype controls. Representative image of laminin-stained tibialis anterior muscle analyzed with CSA macro on ImageJ. While mean myofiber CSA is decreased, Ames mice carry more myofibers/muscle than wildtype controls. A few individuals from each genotype were measured - full cohort not analyzed.
